# Amino Acid Substitutions in the *Caenorhabditis elegans* RNA Polymerase II Large Subunit AMA-1/RPB-1 that Result in α-Amanitin Resistance and/or Reduced Function

**DOI:** 10.1534/g3.111.000968

**Published:** 2011-11-01

**Authors:** Elizabeth Anne Bowman, Donald L. Riddle, William Kelly

**Affiliations:** * Department of Biology and; † Biochemistry, Cell and Developmental Biology Program, Emory University, Atlanta, Georgia 30322; ‡ Michael Smith Laboratories, University of British Columbia, Vancouver, British Columbia V6T1Z4, Canada

**Keywords:** *Caenorhabditis elegans*, RNA polymerase II, AMA-1/RPB-1, α-amanitin

## Abstract

Mutations in the *Caenorhabditis elegans* RNA polymerase II AMA-1/RPB-1 subunit that cause α-amanitin resistance and/or developmental defects were isolated previously. We identified 12 of these mutations and mapped them onto the *Saccharomyces cerevisiae* RPB1 structure to provide insight into AMA-1 regions that are essential for development in a multicellular organism.

The DNA-directed RNA polymerase II (Pol II) holoenzyme is a ∼500 kDa complex responsible for transcribing protein-coding and other genes in eukaryotes. The Pol II complex is composed of 12 subunits that are highly conserved from yeast to human (supporting information, Figure S1). The largest subunit, RPB1, is a ∼200 kDa multidomain protein that makes up much of the functional core of Pol II (Cramer *et al.* 2001). During transcription, dsDNA enters between the two lobes of the Pol II jaw-like structure, largely contacting the cleft domain of the RPB1 protein. At the base of the jaws lies the active site domain of RPB1. Although this is the domain primarily responsible for RNA polymerization, components of both the cleft and funnel domains are required for transcription as they allow translocation of the template DNA within the Pol II complex. Specifically, the trigger loop, located outside of the active site, is essential for proper catalysis by monitoring substrate selectivity as ribonucleotides pass through the RPB1 pore domain to the active site (Kaplan *et al.* 2008). In addition, interactions between the trigger loop and the bridge helix, an a-helix that spans the Pol II jaws, are thought to mediate the flexibility of the bridge helix that is important for translocation of the duplex DNA (Brueckner and Cramer 2008; Wang *et al.* 2006).

Understanding the mechanism of Pol II transcription has been aided by specific inhibitors of this complex. The “death cap” mushroom toxin, α-amanitin, prevents transcriptional elongation in most eukaryotes by sterically blocking the intramolecular interactions between the trigger loop and bridge helix required for translocation (Brueckner and Cramer 2008; Kaplan *et al.* 2008; Wang *et al.* 2006). Mutations that confer α-amanitin resistance are mostly located in the “funnel” domain of RPB1, a region close to the active site of the enzyme that contains the trigger loop (Bushnell *et al.* 2002).

Studies in *Caenorhabditis elegans* were among the first to characterize mutations in the worm gene, *ama-1/pb-1*, and their effects on both α-amanitin binding and developmental processes (Rogalski *et al.* 1988; Rogalski *et al.* 1990; Rogalski and Riddle 1988). These studies mapped mutations within the *ama-1* gene but did not identify the specific DNA changes mutations (Bullerjahn and Riddle 1988). The original collection of mutations represents a variety of *ama-1* alleles, including α-amanitin–resistant, hypomorphic, and putative null alleles. To further define the nature of these alleles, we sequenced a number of the original mutations and mapped them onto the highly homologous *Saccharomyces cerevisiae* Pol II structure to provide potential structure-function information for these regions of the protein. We show that mutations that disrupt susceptibility to α-amanitin lie within the toxin binding site and that one of these also disrupts Pol II function at elevated temperatures. Furthermore, we identify mutations in conserved regions of the protein that cause significant alterations in RNA polymerase II function and provide further insight into its transcriptional mechanism.

## Materials and Methods

### Strains

The following mutations and balancers were used: wild-type N2 (Bristol); LG IV, V: *ama-1(m322)*; *ama-1(m118m526)*; *ama-1(m118)dpy-13(e184)*; *ama-1(m118m251)dpy-13(e184)*; *ama-1(m118m238)dpy-13(e184)*; *ama-1(m118m367m414)dpy-13(e184)*; *ama-1(m118m396)dpy-13(e184)IV/nT1,V/nT1*; *ama-1(m118m235)dpy-13(e184)IV/nT1,V/nT1*; *ama-1(m118m236)dpy-13(e184)IV/nT1,V/nT1*; *ama-1(m118m332)dpy-13(e184)IV/nT1,V/nT1*; *ama-1(m118m370)dpy-13(e184)IV/nT1,V/nT1*; *ama-1(m118m370m417)dpy-13(e184)IV*; and *ama-1(m118m367)dpy-13(e184)IV/nT1,V/nT1*.

### Sequencing *ama-1* mutations

Two to four kilobytes of overlapping fragments of *ama-1* were amplified by PCR from 10 animals of each genotype using high-fidelity Phusion polymerase (Finnzymes) and sequenced (Macrogen USA Sequencing). Sequencing covered the entire coding region, including introns and exons. The sequence was compared to the published wild-type F36A4.7 (*ama-1*) sequence on http://www.wormbase.org. For any mutation identified, the corresponding *ama-1* fragment was independently amplified and sequenced to confirm that the mutation was not due to the amplification step. For mutant strains with early arrest phenotypes (DR811, DR880, DR877), heterozygous animals from balanced strains were used for amplification and sequencing, and a mutation was identified as a heterozygous (double) peak in the sequencing chromatogram. For any mutation identified in a balanced strain, the corresponding *ama-1* fragment from homozygous *ama-1* mutant worms [marked by *dpy-13(e184*)] were amplified and sequenced.

### Alignment to AMA-1/RPB-1 structures

To identify the possible structural alterations that lead to the observed phenotypes in *ama-1* mutants, the amino acids altered in *C. elegans ama-1* mutants were mapped to homologous amino acids in the *S. cerevisiae* RNA polymerase II structure. Specifically, the *C. elegans* AMA-1 and *S. cerevisiae* RPB1 amino acid sequences were aligned using Clustal W (Thompson *et al.* 1994). *S. cerevisiae* amino acids homologous to *C. elegans* mutations were identified in one of two RNA polymerase II structures: PMID 3cqz (Kaplan *et al.* 2008) for analysis of α-amanitin-resistant mutations and PMID 2vum (Brueckner and Cramer 2008) for hypomorphic mutations.

### Assaying temperature-sensitive phenotypes

Worms were either maintained at 16° or shifted from 16° to 25° at either the L1 or L4 larval stage, and the phenotypes of their progeny were recorded. The total number of embryos produced by each animal and laid on the plates was counted to determine brood size. Embryonic lethality was recorded as the percentage of unhatched embryos after 24 hr. To assay gastrulation, embryos were probed using rabbit anti-PGL-1, which labels the primordial germ cells Z2/Z3, and DAPI to stain DNA as previously described (Schaner *et al.* 2003). Embryos were scored as gastrulation defective if Z2/Z3 were found to remain among the external layer of embryonic nuclei (Powell-Coffman *et al.* 1996).

## Results

A large, valuable collection of mutant *C. elegans* worms with changes in the RNA polymerase II large subunit gene *ama-1/rpb-1* was generated by EMS mutagenesis in the 1980s. This represents the second largest collection of metazoan mutants in *ama-1/rpb-1*, yet the identification of the corresponding changes in the *ama-1* gene that result in these defects have remained unexplored. We have thus revisited this resource to further characterize these *ama-1* mutations and to investigate their possible structural effects.

### α-Amanitin–resistant mutations

**Figure 1:**
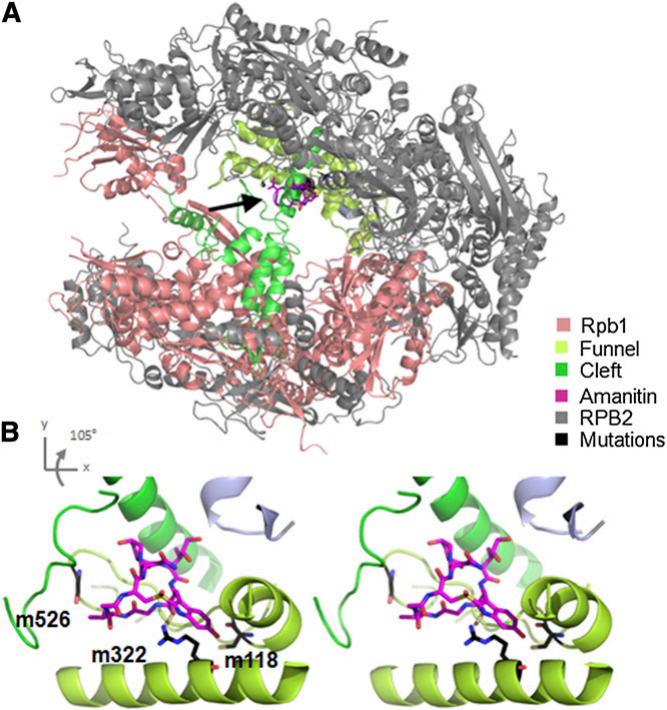
Structural position of *C. elegans* α-amanitin–resistant mutations in the homologous *S. cerevisiae* structure. (A) Location of α-amanitin binding site in the *S. cerevisiae* Pol II structure (PDB ID: 3cqz) (Kaplan *et al.* 2008). α-amanitin (arrow) binds to the AMA-1/RPB-1 subunit between the “funnel” and “cleft” domains (light olive and bright green domains, respectively). (B) Stereo image of the location of amino acids corresponding to *C. elegans* α-amanitin resistance-inducing mutations. All amino acids changes are within the α-amanitin binding site and are conserved from yeast to *C. elegans*. The amino acid corresponding to m322 makes a hydrogen bond with α-amanitin. Figure rendered in PyMOL (The PyMOL Molecular Graphics System, Version 1.3, Schrödinger, LLC, http://www.pymol.org).

α-Amanitin binds AMA-1/RPB-1 and blocks transcriptional elongation by preventing AMA-1/RPB-1 trigger loop–mediated substrate selection and bridge helix flexibility during translocation (Brueckner and Cramer 2008; Bushnell *et al.* 2002; Kaplan *et al.* 2008; Wang *et al.* 2006). We sequenced two previously isolated mutants in the *C. elegans ama-1* gene that demonstrated α-amanitin resistance (Rogalski *et al.* 1988; Rogalski *et al.* 1990; Rogalski and Riddle 1988). For one allele, *ama-1(m118*), we verified a mutation previously identified as a C777Y substitution (D. M. Bird and D. L. Riddle, unpublished). We also identified a novel *C. elegans* α-amanitin–resistant mutation, *ama-1(m322*), as an R739H substitution (Table 1). Mapping these *ama-1* mutations onto the *S. cerevisiae* structure of RPB1 showed that both mutations were in the trigger loop of the RPB1 “funnel” domain (Figure 1). The arginine residue in yeast that is homologous to R739 in AMA-1, R726, provides a hydrogen bond with α-amanitin, suggesting that R739 performs a similar function in *C. elegans* (Figure 1B, Table 1) (Bushnell *et al.* 2002). An R to H change in the *C. elegans* protein would alter the distance critical for this hydrogen bond in the α-amanitin binding pocket and weaken this interaction. An identical substitution in the corresponding amino acid in *Drosophila* and in mouse cells has also been shown to inhibit α-amanitin binding [RpbII215-4; R741H (Chen *et al.* 1993; Coulter and Greenleaf 1982; Greenleaf *et al.* 1979); RpII215-A21, R749P, (Bartolomei and Corden 1995)]. Interestingly, the corresponding mutation in yeast suppresses a transcription start site defect [sit1-290, (Archambault *et al.* 1998)].

**Table 1:** Summary of α-amanitin–resistant and hypomorphic *ama-1* mutations

Strain *^a^*	Allele	DNA Mutation	AA Change	Corresponding *S. cerevisiae* AA	Location in Structure *^b^*	Terminal Phenotype *^c^*	
						20°	25°
N2		N. M. *^d^*					
DR680 *^e^*	m118	3893 g→a	777 C→Y	764 C	Funnel, near α-amanitin binding site	Adult (F)	Adult (F)
DR786 *^e^*	m322	3481 g→a	739 R→H	726 R	Funnel, near α-amanitin binding site	Adult (F)	Adult (F)
DR1099 *^e^*	m118	3893g→a	777 C→Y	764 C	Funnel, near α-amanitin binding site	Adult (F)	Adult (ME)
	m526	3917 g→a	785 G→E	772 G	Funnel, near α-amanitin binding site		
DR731 *^f^*	m118	3893g→a	777 C→Y	764 C		Adult (F)	Adult (ME)
	m251	1719 c→t	363 A→V	355 G	Active site, α-helix 8, between rpb1/2		
DR730 *^f^*	m118	3893g→a	777 C→Y	764 C		Adult (F)	Adult (ME)
	m238	6623 g→a	1406 G→R	1388 G	Cleft, loop		
DR892 *^f^*	m118	3893 g→a	777 C→Y	764 C		Adult (ME)	Mid larval arrest
	m396	6414 c→t	1336 S→F	1318 T	Cleft, β-sheet 44, between rpb1/5		
DR682 *^f^*	m118	3893g→a	777 C→Y	764 C	Cleft (trigger loop), β-sheet 36	Mid larval to adult (ME) arrest	Mid larval arrest
	m235	5210 g→a	1086 G→E	1073 G			
DR683 *^f^*	m118	3893g→a	777 C→Y	764 C		Adult (ST)	L1 larval arrest
	m236	5105 a→t	1051 N→I	1038 T	Foot, β-sheet 35		
DR811 *^g^*	m118	3893g→a	777 C→Y	764 C		L1 larval arrest	L1 larval arrest
	m332	4168 g→a	869 V→M	856 T	Cleft, α-helix 24		
DR880 *^g^*	m118	3893g→a	777 C→Y	764 C		L1 larval arrest	L1 larval arrest
	m370	3171 g→a	636 G→R	623 G	Pore 1, β-sheet 17		
DR976 *^h^*	m118	3893g→a	777 C→Y	764 C		Adult (F)	Adult (F)
	m370	3171 g→a	636 G→R	623 G			
	m417	3502 c→t	746 A →V	733 A	Funnel, β-sheet 21		
DR877 *^g^*	m118	3893g→a	777 C→Y	764 C		L1 larval arrest	L1 larval arrest
	m367	5282 g→a	1110 G→E	1097 G	Cleft (trigger loop), β-sheet 37		
DR966 *^i^*	m118	3893g→a	777 C→Y	764 C		Adult (F)	Adult (F)
	m367	N. M. *^d^*					
	m414	N. M. *^d^*					

Numbering of the DNA sequence is from the genomic sequence, beginning at the translation start site. All DNA mutations that were identified were found within exons; no mutations within introns were observed. The presumed amino acid change that would result from the DNA mutation is indicated. The *C. elegans* and *S. cerevisiae* AMA-1/RPB-1 amino acid sequences were aligned using Clustal W (Thompson *et al.* 1994), and the homologous *S. cerevisiae* amino acid corresponding to the mutated residues in each strain are indicated.

a Isolated previously. DR680, DR786, DR683, DR682, DR730, and DR731 (Rogalski and Riddle 1988); DR1099 (Rogalski *et al.* 1990); and DR892, DR811, DR880, DR976 DR877, DR966 (Rogalski *et al.* 1988).

b Domain and secondary structure specified as in Cramer *et al.* (2001).

c Phenotypes characterized previously by Rogalski *et al.* (1988). ME, maternal effect embryonic lethal; ST, does not lay eggs; F, fertile, producing 70–90 progeny. L1 larval arrest is a null phenotype: this phenocopies the terminal phenotype an *ama-1* deletion allele, which is L1 arrest (Rogalski and Riddle 1988).

d No mutation.

e α-Amanitin–resistant.

f Hypomorphic *ts*-mutant.

g Null mutant.

h Rescue of DR880 phenotype.

i Rescue of DR877 phenotype.

We also identified the mutations in an α-amanitin “super-resistant” strain [DR1099: *ama-1(m118m526*)] that was isolated after further mutagenesis of the *ama-1(m118*) strain (Rogalski *et al.* 1990). The *ama-1* gene in this strain carries a second mutation which creates a G785E transition which we predict to sterically block the α-amanitin binding pocket in the protein structure. Alteration of the corresponding amino acid also inhibits α-amanitin binding in the mouse protein (Bartolomei and Corden 1995).

In addition to the high resistance to α-amanitin, DR1099 displays temperature-sensitive defects consistent with defective RNA Pol II function (Rogalski *et al.* 1990). *ama-1(RNAi*) embryos exhibit gastrulation defects and arrest at ∼120 cells (Powell-Coffman *et al.* 1996). Similarly, when DR1099 animals are shifted to a restrictive temperature (25°), their embryos fail to gastrulate and arrest at ∼120 cells (Table 2). Importantly, however, immunofluorescence analyses of these embryos detected significant levels of an epitope that correlates with the elongating form of RNA Pol II, phosphorylation of Ser2 on the CTD repeat peptide [(Saunders *et al.* 2006); data not shown]. Thus, whereas RNA Pol II function is not completely compromised at the restrictive temperature, elongation processivity may be significantly affected, perhaps by a temperature-dependent mechanism that mimics inhibition of elongation by α-amanitin. This seems likely when considering the potential impact of G785E on the predicted structure. The bulky and charged E side-chain could insert within and disrupt the interface between the trigger loop and bridge helix of AMA-1 (Figure 1B). It is unclear, however, whether the effects of the G785E substitution are autonomous or exist only in the context of the second *m118* C777Y substitution.

**Table 2:** DR1099 phenotype characterization

	16° (n = 8)	Shifted as L1, 25° (n = 7)	Shifted as L4, 25° (n = 7)
Brood size	174.5 ± 44.4	31.0 ± 16.1	80.6 ± 34.3
Embryonic lethality	0%, n = 1396	95.4%, n = 217	98.8%, n = 564
Gastrulation defective	No	Yes	Yes

To measure a temperature sensitive defect in transcription, embryonic lethality and gastrulation was assayed in DR1099 worms maintained at the permissive temperature, 16°, or shifted to the restrictive temperature, 25°, as L1s or L4s.

Interestingly, residues that when altered confer α-amanitin resistance in *C. elegans* are also changed in the *Giardia lamblia* *rpb1* gene, which is naturally α-amanitin resistant. The positions in *Girardia rbp1* that correspond to those in *C. elegans ama-1*, and the amino acid differences between the species, are as follows: *Ce* R739 = *Gl* S851, *Ce* C777 = *Gl* S889, and *Ce* G785 = *Gl* S897 (Seshadri *et al.* 2003).

### Hypomorphic and null mutations

**Figure 2:**
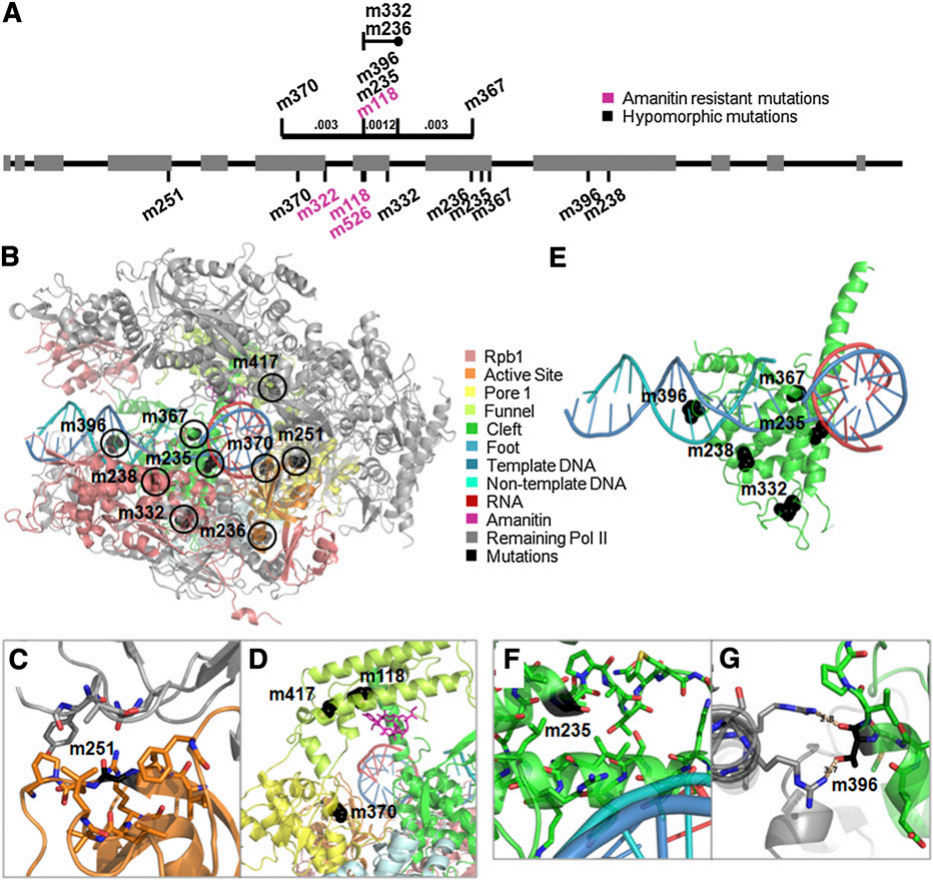
Positions of the *C. elegans* AMA-1/RPB-1 mutations mapped to corresponding residues in the homologous *S. cerevisiae* structure. (A) Comparison of location of mutations along the *ama-1* sequence (exons in gray boxes) with previous fine-structure genetic map position (Bullerjahn and Riddle 1988). (B) Structural location of hypomorphic and null mutations in the Pol II structure (PDB ID: 2vum) (Brueckner and Cramer 2008). Domains and domain-like regions are identified according to Cramer *et al.* (2001); mutations are identified by their allele names. See Table 1 for allele descriptions. (C) Structural location of m251 in RPB-1/RPB-2 binding face. (D) Structural location of mutations found in DR976: m118, α-amanitin resistance mutation; m370, null mutation; and m417, rescue mutation. Mutations m370 and m417 are approximately 27Å apart. (E) Structural location of mutations within cleft domain and DNA binding domain. (F) Structural location of m235 in the cleft “trigger loop” and proximity to bridge helix (bottom α-helix). (G) Structural location of m396 and hydrogen bonds to RPB5 R11 and R14 in 3cqz structure (Kaplan *et al.* 2008). Bond distance is indicated. Figure rendered in PyMOL (http://www.pymol.org).

Recessive-lethal alleles of *ama-1* were also isolated through further mutagenesis of the *ama-1(m118*) strain. (Rogalski *et al.* 1988; Rogalski and Riddle 1988). The phenotypes of these mutants range from temperature-sensitive (*ts*) sterile (presumed hypomorphic allele) to L1 arrest (presumed null allele; *i.e.*, phenocopies *ama-1* deletion alleles). To better correlate Pol II structural alteration with phenotype, we sequenced ten of these mutants and mapped them onto the *S. cerevisiae* Pol II structure (Table 1, Figure 2A, B). The following mutations represent alterations in residues and domains that are highly conserved among yeast, worms, flies, and humans (see Figure S1 for AMA-1 sequence alignment).

The *ama-1(m370*) mutation G636R, which yields a null-function phenotype, affects a residue predicted to lie at the cap of a beta sheet in the “pore” domain of AMA-1. This amino acid is in a tightly packed region of the Pol II protein, and the dramatic G to R residue change likely disrupts this packing and thus indirectly disrupts catalysis (Figure 2D).

The *ama-1(m370*) strain DR880 [*ama-1(m118m370*)] was further mutagenized to identify suppressors of the *ama-1(m370*) lethal phenotype (Rogalski *et al.* 1988). We identified the presumed suppressing mutation in one of these strains, DR976: *ama-1(m118m370m417*), as an A746V conversion. Surprisingly, upon placement onto the yeast structure, the positions of G636 [*ama-1(m370*) mutation] and A746 [*ama-1(m417*) mutation] are predicted to lie at least 27Å apart within two different domains of AMA-1/RPB-1 (Figure 2D). G636 is predicted to lie within the pore domain, and A746 within the distant funnel domain. Further outcross experiments confirmed the very tight linkage of *ama-1(m370*) and *ama-1(m417*), supporting the conclusion that the suppression phenotype is indeed caused by the A746V conversion.

A similar long-distance effect has been observed in *Drosophila*. The RpII215^K1^ mutation (*D.m*. S678N, *S.c*. S663) is found within the pore domain of *Drosophila* RPB-1 and causes a *ts* phenotype. This phenotype can be rescued by two different intragenic mutations in the funnel domain (RpII215^R4^: *D.m*. H713L, *S.c*. 698-Q; RpII215^R10^: *D.m*. S747L, *S.c*. 732-L), which are 49Å and 24Å away from RpII215^K1^, respectively (Chen *et al.* 1993; Krasnoselskaya *et al.* 1998; Mortin *et al.* 1988). Sequences of more internal revertants of *ama-1* hypomorphs might reveal how common it is for intragenic revertants to exert their effects over long distances.

The remaining five mutations identified in this study that are highly conserved residues all lie within the “cleft” domain of AMA-1/RPB-1 (Figure 2E) (Cramer *et al.* 2001). This domain makes up a large portion of the DNA binding region of the lower “jaw” of Pol II. The *ama-1(m332*) mutation V869M, which results in a null phenotype, corresponds to a residue in the *S. cerevisiae* structure that lies within a densely packed region of Pol II. While this particular amino acid is not conserved in yeast, it is in a very well conserved domain, and changing the small hydrophobic valine to a larger, more hydrophilic methionine may cause clashing with nearby side chains and disrupt this packing. The *ama-1(m235*) and *ama-1(m367*) mutants (G1086E and G1110E, respectively) correspond to yeast positions that are both found in the trigger loop of the cleft domain. This domain is thought to couple nucleotide recognition and catalysis during Pol II translocation, and substitutions at either glycine could decrease flexibility of the catalytically important bridge helix (Kaplan *et al.* 2008; Wang *et al.* 2006). The *ama-1(m235*) mutation is also in close proximity to Q838 in the bridge helix (Figure 2F). This contact is likely important because the sequence of the entire bridge helix is highly conserved, so the glycine to glutamate is presumably poorly tolerated. The G1110 residue does not contact the bridge helix directly, but it is also in a well-packed region of AMA-1. Conversion of G1110 to E in *ama-1(m367*) presumably disrupts packing in this domain. An EMS-induced revertant of *ama-1(m367*) (DR877) contained only the *ama-1(m118*) α-amanitin–resistant mutation; the E1110 substitution had been converted back to glycine.

The *ama-1(m238*) mutation G1406R results in temperature-sensitive, maternal-effect, embryonic-lethal phenotype. G1406 is predicted to be in the “switch 1 domain” of the cleft domain, which is thought to be important for the Pol II conformational changes that allow template binding (Gnatt *et al.* 2001). Mutation of this amino acid in yeast causes cold sensitivity and slow growth by affecting transcription start site selection (Berroteran *et al.* 1994; Hampsey *et al.* 1991). The *ts* phenotype in *C. elegans* may involve a similar mechanism.

The amino acid substitution in *ama-1(m396*), S1336F, is also found in the cleft domain and is specifically in the binding interface between RPB1 and RPB5 (Figure 2G). In yeast, the corresponding amino acid T1318 is predicted to have ionic interactions with R11 and R14 of the RPB5 subunit. This binding is likely conserved in *C. elegans* as this side-chain hydroxyl and the RPB-5 arginine residues are conserved (RPB-5 R10 and R13 in *C. elegans*), and mutation of this amino acid could disrupt RPB-1/5 interactions.

Two mutations identified by sequencing resulted in amino acid substitutions in positions where there was little conservation. The *ama-1(m251*) mutation A363V has a temperature-sensitive, maternal-effect, embryonic-lethal phenotype. A363 corresponds to a glycine amino acid in yeast and a proline in flies and humans. Although the residue itself is not conserved, the surrounding residues are highly conserved among all four species, and it is located within the active site domain of yeast RPB-1 (Figure 2C). Although this mutation results in a conservative amino acid change, A363 is also predicted to lie within the RPB-1/RPB-2 interface, and disruption of this interaction may explain its *ts* phenotype. However, how a conservative change in *C. elegans* can affect AMA-1 function and yet maintain the normal protein as a proline in flies and humans is not understood.

The *ama-1(m236*) mutation N1051I, which results in sterility and *ts* larval arrest, is predicted to lie within the AMA-1/RPB-1 “foot” domain. The asparagine in *C. elegans* is a significant alteration relative to the other species, which have either serine (yeast) or threonine (fly and human). The surrounding amino acids are also significantly diverged, although this domain serves as a binding site for the yeast mRNA capping enzyme, CE (Suh *et al.* 2010). The yeast amino acid position corresponding to N1051 [T1038] lies on the edge of the CE electron density in the yeast Pol II–CE cocrystal (Suh *et al.* 2010). It is likely that much of the structure of this region is devoted to the proper presentation of important/conserved residues for this interaction. Thus the N to I substitution might result in temperature-dependent instability in the interaction between the *C. elegans*–capping enzyme and AMA-1.

## Discussion

Sequencing these previously identified mutants of *C. elegans* *ama-1* helps to complete mutagenesis studies done over 20 years ago. Overall, the positions of the mutations identified by sequencing closely match their positions originally determined by fine-structure genetic mapping (Figure 2A). The genetic results thus provide strong supporting data to conclude that the nucleotide changes identified are causative for the functional phenotypes observed in these mutants.

We identified two novel *C. elegans* α-amanitin–resistant mutations. One of these mutations, *ama-1(m526*), confers a tight *ts*, maternal-effect, lethal phenotype that may result from reduced efficiency of elongation, and it could be useful in further studies of Pol II function. In addition, assigning the structural positions of null and hypomorphic mutations may provide important structure-function clues for understanding how these mutations lead to different functional consequences that have biologic read-outs as different developmental phenotypes. Further biochemical analyses on the structure-function relationship of the mutations isolated from these early genetic screens will provide new information about the Pol II structure, and this emphasizes the mutual benefits that combined genetic and biochemical-structural approaches can provide. This article should serve as a valuable community resource for those seeking to understand such structure-function relationships, as well as those seeking to employ defined *ama-1* mutations in their studies.

## Supplementary Material

Supporting Information
