# Supplementary material for: Amino Acid Substitutions in the Caenorhabditis elegans RNA Polymerase II Large Subunit AMA-1/RPB-1 that Result in α-Amanitin Resistance and/or Reduced Function
Source: G3 (Bethesda). 2011 Nov 1;1(6):411–6. doi: 10.1534/g3.111.000968 (PMC3276164; doi:10.1534/g3.111.000968)
Supplement: Supporting Information [file supp_1_6_411__index.html]

Supporting Information 

# Amino Acid Substitutions in the *Caenorhabditis elegans* RNA Polymerase II Large Subunit AMA-1/RPB-1 that Result in α-Amanitin Resistance and/or Reduced Function

## Supporting Infomation for FBowman, Riddle, and Kelly, 2011

**Files in this Data Supplement:**

- Figure S1 - Sequence alignments of *Saccharomyces cerevisiae* (y), *Caenorhabditis elegans* (c), *Drosophila melanogaster* (d), and *Homo sapiens* (h) AMA-1/RPB-1 amino acid sequences (PDF, 96 KB)
