# Supplementary material for: Amino Acid Substitutions in the Caenorhabditis elegans RNA Polymerase II Large Subunit AMA-1/RPB-1 that Result in α-Amanitin Resistance and/or Reduced Function
Source: G3 (Bethesda). 2011 Nov 1;1(6):411–6. doi: 10.1534/g3.111.000968 (PMC3276164; doi:10.1534/g3.111.000968)
Supplement: Supporting Information [file supp_1.6.411_FigureS1.pdf]

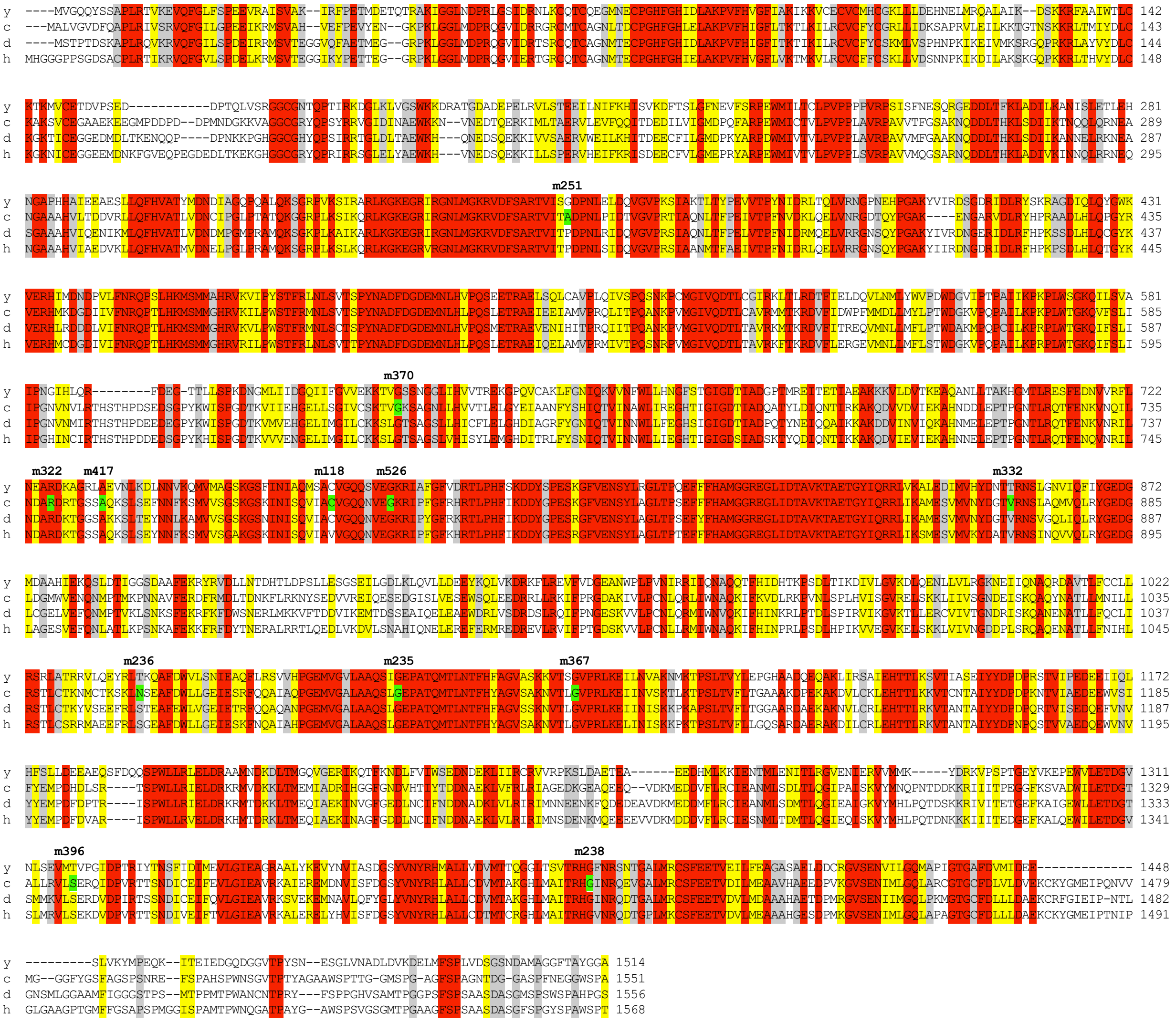

Figure S1 Sequence alignments of *Saccharomyces cerevisiae* (y), *Caenorhabditis elegans* (c), *Drosophila melanogaster* (d), and *Homo sapiens* (h) AMA-1/RPB-1 amino acid sequences. *C. elegans* mutations identified in this study are highlighted in green. Sequence conservation indicated by red (identical), yellow (conserved), and grey (semi-conserved) highlights. Alignment done in Clustal W (Thompson *et al.* 1994).

Thompson, J. D., D. G. Higgins and T. J. Gibson, 1994 CLUSTAL W: improving the sensitivity of progressive multiple sequence alignment through sequence weighting, position-specific gap penalties and weight matrix choice. Nucleic Acids Res **22:** 4673-4680.
